# Population Pharmacokinetics of Trametinib and Impact of Nonadherence on Drug Exposure in Oncology Patients as Part of the Optimizing Oral Targeted Anticancer Therapies Study

**DOI:** 10.3390/cancers16122193

**Published:** 2024-06-11

**Authors:** Anne Ravix, Carole Bandiera, Evelina Cardoso, Adrian Lata-Pedreira, Haithem Chtioui, Laurent Arthur Decosterd, Anna Dorothea Wagner, Marie Paule Schneider, Chantal Csajka, Monia Guidi

**Affiliations:** 1 Centre for Research and Innovation in Clinical Pharmaceutical Sciences, Lausanne University Hospital and University of Lausanne, 1011 Lausanne, Switzerland; 2 Institute of Pharmaceutical Sciences of Western Switzerland, University of Geneva, 1211 Geneva, Switzerlandmarie.schneider@unige.ch (M.P.S.); 3 School of Pharmaceutical Sciences, University of Geneva, University of Lausanne, 1211 Geneva, Switzerland; 4 Centre for Primary Care and Public Health (Unisanté), University of Lausanne, 1011 Lausanne, Switzerland; 5 Service of Clinical Pharmacology, Lausanne University Hospital and University of Lausanne, 1011 Lausanne, Switzerland; 6 Laboratory of Clinical Pharmacology, Lausanne University Hospital and University of Lausanne, 1011 Lausanne, Switzerland; 7 Department of Oncology, Lausanne University Hospital and University of Lausanne, 1011 Lausanne, Switzerland

**Keywords:** trametinib, oral anticancer therapy, population pharmacokinetics, simulations, medication adherence

## Abstract

**Simple Summary:**

Poor adherence to trametinib, an oral anticancer drug, may be the consequence of side effects that severely impact the patient’s quality of life. The significant interindividual variability associated with poor adherence results in suboptimal drug exposure and consequently in unfavourable patient outcomes. By characterizing the pharmacokinetics of trametinib, this study aims to assess (i) the adequacy of recommended doses to achieve efficacy thresholds and (ii) the impact of non-adherence on drug exposure. The latter was assessed by simulating different scenarios of missing one or more doses per week to highlight the risk of treatment failure associated with poor adherence. These results promote interprofessional collaboration and patient partnership to address patients’ needs in order to ensure adherence to trametinib and *in fine* therapeutic success.

**Abstract:**

Trametinib is a targeted therapy used for the treatment of solid tumours, with significant variability reported in real-life studies. This variability increases the risk of suboptimal exposure, which can lead to treatment failure or increased toxicity. Using model-based simulation, this study aims to characterize and investigate the pharmacokinetics and the adequacy of the currently recommended doses of trametinib. Additionally, the simulation of various suboptimal adherence scenarios allowed for an assessment of the impact of patients’ drug adherence on the treatment outcome. The population data collected in 33 adult patients, providing 113 plasmatic trametinib concentrations, were best described by a two-compartment model with linear absorption and elimination. The study also identified a significant positive effect of fat-free mass and a negative effect of age on clearance, explaining 66% and 21% of the initial associated variability, respectively. Simulations showed that a maximum dose of 2 mg daily achieved the therapeutic target in 36% of male patients compared to 72% of female patients. A dose of 1.5 mg per day in patients over 65 years of age achieved similar rates, with 44% and 79% for male and female patients, respectively, reaching the therapeutic target. Poor adherence leads to a significant drop in concentrations and a high risk of subtherapeutic drug levels. These results underline the importance of interprofessional collaboration and patient partnership along the patient’s journey to address patients’ needs regarding trametinib and support medication adherence.

## 1. Introduction

Cancer remains a significant worldwide health issue, with an estimated 20 million new cases reported in 2022 [1]. The incidence of cancer has risen in recent years, primarily due to factors such as an aging population and increased exposure to specific risk factors, including smoking and obesity [2]. Despite the rise in the number of cases, advances in oncological treatments and early screening and detection have significantly improved survival rates and reduced cancer-related mortality [3,4,5]. An important development in this field is the introduction of oral anticancer medications that have reduced the burden of treatment [6,7]. Patients have increased autonomy in managing their medication, enabling them to receive treatment in the comfort of their own environment and to reduce the need for hospital visits [8]. Oral therapies also offer greater flexibility [9]. Targeted therapies, a common form of treatment, work by inhibiting the signalling pathways responsible for the development of cancer cells [10]. Compared to chemotherapy, these therapies have fewer adverse effects on normal cells and target cancer cells more efficiently. However, a number of disadvantages associated with oral administration have been identified, such as variations in absorption due to administration with meals or at irregular times, and the risk of interactions with other medications [6,8,11,12]. The variability in adherence to treatment is also a major concern, as 46% to 100% of patients take their medications as prescribed [13]. There are several reasons for nonadherence, including side effects, daily administration that can be difficult to fit into a patient’s schedule, the conditions of the intake (e.g., fasting conditions), and the length of the treatment, which can lead to patient fatigue and invisible results [14]. The outpatient care reduces contact with healthcare professionals, and there may be less opportunity for patients to report their concerns regarding their medications, which sometimes results in patients temporarily discontinuing their treatments without informing their prescribers. Such undesired interruptions contribute to treatment failure [15]. An approach to optimize patient medication adherence may be their inclusion into a tailored medication adherence program associated with therapeutic drug monitoring (TDM). The TDM allows for last dose intake to be monitored by checking plasma concentrations, but can also adjust individual doses by managing variability to optimize efficacy and reduce side effects [16]. This tailored approach was the main objective of the Optimizing oral Targeted Anticancer Therapies (OpTAT), ClinicalTrials.gov: NCT04484064 study, conducted in collaboration between the University hospital of Lausanne and the Centre of Primary Care and Public Health (Unisanté) in Lausanne [17].

**Figure 1:**
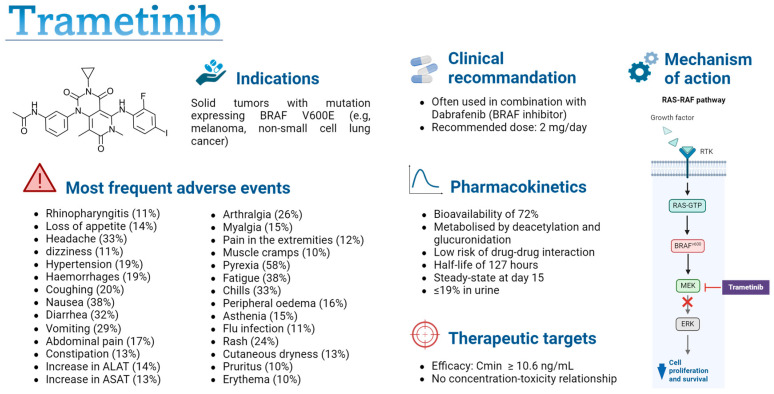
Summary of trametinib characteristics [18,19,20,33,34,35,40]. C_min_: minimal concentration; PK-PD: pharmacokinetic–pharmacodynamic; RTK: receptor tyrosine kinase; MEK: mitogen-activated protein kinase; ERK: extracellular regulated kinases. Figure created with permission from Biorender.com.

Numerous tyrosine kinase inhibitors (TKIs) have been developed in recent years, and further studies are needed to optimize clinical recommendations, which often advocate single doses despite significant pharmacokinetic (PK) variability [7,18]. This study will focus on trametinib, which is widely used in clinical practice for the treatment of melanoma, for which a PK–efficacy relationship has been observed [18]. Trametinib is a targeted therapy for the treatment of solid tumours in patients with the V600 mutation in the BRAF gene, which encodes a serine/threonine protein kinase that leads to the activation of the mitogen-activated protein kinase/extracellular-regulated kinase (MAPK/ERK) signalling pathway (see Figure 1) [19]. This mutation stimulates uncontrolled cell proliferation and promotes the development of cancer cells. Trametinib is a reversible, selective, allosteric inhibitor of mitogen-activated protein kinases (MEK) 1 and 2 activation, which blocks the activation of the ERK signalling pathway, thus preventing cancer cell proliferation [19,20]. The use of trametinib as a monotherapy or in combination with dabrafenib, another BRAF-targeted inhibitor, improves progression-free survival compared to treatment with chemotherapy or other oral anticancer agents [21,22,23,24,25,26,27]. The combination of dabrafenib and trametinib is used as a first-line treatment in BRAF-mutant non-small cell lung cancer and as a first- or second-line treatment in BRAF-mutant melanoma [28,29,30,31]. A recent meta-analysis comparing the side effects of three BRAF/MEK inhibitors (Dabrafenib/Trametinib, Vemurafenib/Cobimetinib, and Encorafenib/Binimetinib) revealed similar toxicity, suggesting that therapy should be selected based on patient history to avoid specific side effects in patients already suffering from chronic diseases affecting the same organs (e.g., rheumatological pathologies, hepatopathy, liver dysfunctions, cardiovascular diseases) [32]. Currently, it is administered at a daily dose of 2 mg [33,34]. The drug is rapidly absorbed, with a 72% bioavailability, and maximum concentration is reached approximately 1.5 h after administration [33,35]. Its metabolism occurs primarily through deacetylation, with or without monooxygenation, and it is not a substrate of cytochrome P (CYP) enzymes, lowering the risk of drug–drug interactions [33]. The drug’s terminal half-life is around 127 h, and only 19% is excreted in the urine [33]. Significant variability in PK has been observed in real life studies and can be caused by factors such as bodyweight, age, and food intake [36,37,38]. This variability increases the risk of suboptimal exposure resulting in treatment failure or in an increased toxicity. Trametinib can cause various adverse effects, such as fever, diarrhoea, fatigue, and nausea, sometimes leading to a pause in treatment for a few days [39]. Meanwhile, these adverse effects increase the risk of treatment discontinuation. This poor adherence could have a considerable impact on the efficacy of trametinib, leading to a decrease in plasma concentrations, potentially bringing them below the efficacy threshold defined for this drug [18].

The aim of this study was to characterize the PK of trametinib and to investigate the influence of demographic and clinical factors on its exposure using real-life data from adults with solid cancer. Model-based simulations were performed to investigate the adequacy of doses currently used in practice in achieving the efficacy threshold and to assess the impact of poor adherence on drug exposure.

## 2. Materials and Methods

### 2.1. Data

Data were collected during the OpTAT study [17]. Patients included adults receiving trametinib for the treatment of their solid tumour (e.g., melanoma, ovarian cancer, breast cancer). Blood samples were collected from patients during their regular medical visits, with a maximum of eight samples taken per patient. To better characterize the PK profile, two patients consented to provide extensive samples, with eight concentrations measured between 0 and 24 h after drug intake. Trametinib plasma levels’ quantification was performed using liquid chromatography coupled with tandem mass spectrometry using an adaptation of the multiplex method developed at the Lausanne University Hospital [41]. The lower limit of quantification and detection for trametinib was 1 and 0.5 ng/mL, respectively.

The effective dose history was integrated into the database for 11 patients, who used a digital bulk pillbox (MEMS and MEMS AS, AARDEX Group) to monitor the date and the time of each opening of the trametinib bottle; these data were cross-checked with information from the adherence interviews reports and the case report forms (“The full adherence information group”) [14]. The digital monitor allowed us to provide accurate information on treatment implementation (i.e., the extent to which the patient’s dosing history is in line with the prescription—the correct dosing regimen, at the correct time and in the right condition) and temporary or long-term discontinuation of treatment (i.e., when the patient stops taking the treatment prematurely) [42]. For the remaining patients, dosing history was reconstructed based on information from the medical consultation notes, assuming steady-state condition if no information was available. Patient demographic and clinical data were extracted from medical records. For all clinical and laboratory data (e.g., body weight, hepatic or renal function), the closest measurement to the blood sample was used. If no measurement was taken within 10 days before or after the sample, then it was considered a missing value. Creatinine clearance (CrCL) was estimated using the Cockcroft–Gault formula [43]. Fat-free mass (FFM) was calculated using the following formula [44]:

(1) $$FFM\left(kg\right)=BW\times \left(1-\frac{{BFP}_{bmi}}{100}\right)$$

with BW denoting bodyweight in kg and $BFP_{bmi}$ denoting body fat percentage, calculated using the following formula:

(2) $$BFP_{bmi}\left(\%\right)=1.20\times BMI+0.23\times AGE-10.8\times SEX-5.4$$

with BMI representing body mass index, in kg/m^2^. SEX = 0 if women and 1 if men and AGE is provided in years.

### 2.2. Modelling and Simulations Analyses

The development of the population pharmacokinetic (popPK) model was performed using nonlinear mixed-effect modelling (Nonmem, ICON, Dublin, Ireland, version 7.4.3) and Pearl speaks Nonmem (PsN, version 4.8.0). Pirana (Certara, Radnor, PA, USA, version 2.9.6) was used for the management of runs, R (version 4.0.2), for graphical and statistical analyses.

#### 2.2.1. Model Building

A classical stepwise procedure was used to build the model. The base model was obtained by comparing the number of compartments, assuming linearity in both trametinib absorption and elimination. Inter-individual variability (IIV) was successively incorporated into each PK parameter. Different error models, including additive, proportional, and combined error models, were also assessed. Different values retrieved from the literature of absorption rate constants (ka), ranging from 0.4 to 2 h^−1^, were evaluated due to insufficient data being collected during the absorption phase [37,38]. For each patient, the time required to reach maximum concentration (Tmax) was estimated. Ka was chosen to provide a Tmax near the reference value of 1.5 h provided by the FDA [40].

Biologically relevant covariates were introduced into the model through a forward/backward insertion/deletion method. Covariates tested were sex, body weight, body mass index (BMI), body surface area (BSA), age, FFM, CrCL, urea, total bilirubin, alkaline phosphatase (PAL), aspartate aminotransferase (ASAT), alanine aminotransferase (ALAT), concomitant administration of dabrafenib, and food intake. Initially, a univariate analysis was conducted, during which covariates were added one by one to the model. Continuous covariates were centred and normalized to the population median and were added to the model using either linear or allometric relationships, while categorical covariates followed a linear relationship. Missing values were assigned the population median. Subsequently, a multivariate analysis was performed of the identified significant covariates to develop the comprehensive model. Finally, a reductive analysis was performed to retain only the most significant covariates.

#### 2.2.2. Sensibility Analysis

The assumption of steady state for patients without adherence information may have biased the analysis by interpreting low levels as high clearances resulting from missed doses rather than from a covariate effect. To verify this, a sensitivity analysis was conducted of the 11 patients with full adherence information. We assumed that if the significant covariate–parameter relationship persisted, its influence on the pharmacokinetic parameter would be confirmed, whereas, if not, this influence might have been confounded by other factors, including suboptimal adherence.

#### 2.2.3. Model Selection

Model selection was based on the difference in objective function values (ΔOFV) between the two nested models, which approximately follows a χ^2^ distribution. Therefore, a decrease of less than 3.84 (*p* < 0.05) was required to select the base model and to retain a covariate in the univariate analysis, and a decrease of less than 6.63 (*p* < 0.01) was required for the reductive analysis. The adequacy of the model’s fit to the data was assessed graphically using goodness-of-fit plots and by checking the relative standard error (RSE) of the parameter estimates.

#### 2.2.4. Model Validation

An internal validation of the final model was performed using the bootstrap method implemented in PsN [45]. Median parameters values with their 95% predictive interval were derived from 2000 replicates of the initial dataset and compared with the original estimates. A prediction-corrected visual predictive check (pcVPC) was also performed using PsN by running 1000 simulations based on the final PK estimates to calculate the median, 5th, and 95th percentiles of the concentration–time profiles [45,46].

#### 2.2.5. Simulations

Simulations based on the final model were performed to verify whether the currently prescribed doses enable the achievement of the therapeutic target, i.e., a minimum concentration (C_min_) greater than or equal to 10.6 ng/mL [18,47,48]. For this, a population of 4000 virtual patients was created, with 1000 patients per sex (male, female) and age category, based on the standard cut-off used to define the older population (<65 years or ≥65 years). The age of each virtual patient was randomly generated from a uniform distribution whose limits depended on the patient’s category, while the fat-free mass was generated from a sex-dependent uniform distribution, whose ranges corresponded to the 10 and 90 percentiles reported in the paper of Larsson et al. [49]. Tested doses ranged from 0.5 to 2 mg, corresponding to the doses used in clinical practice. The comparison of drug exposure between the different dosages was achieved by calculating the percentage of patients reaching the therapeutic target.

Simulations based on the same virtual population were conducted to observe the impact of non-adherence on concentration–time profiles. Several scenarios were tested with a dose of 2 mg every 24 h, including missing only a single dose, missing one or two random doses per week during several weeks, missing two consecutive doses per week, and interrupting treatment for one week and then taking the treatment for two weeks. Each scenario took place at treatment initiation to simulate patients who have difficulty adhering to the trametinib from the beginning, and at steady state to simulate cases where patients start treatment correctly and then become non-adherent, for example, due to treatment fatigue or adverse effects. The single-missed-dose scenario was only evaluated at steady state because skipping doses early in treatment leads to greater uncertainty in drug PK prediction, which further complicates scenarios. The concentration profiles obtained after one or more missed doses were compared with the optimal adherence profile, i.e., without any missed doses.

All simulations were repeated 100 times to obtain a 95% confidence interval for the percentage of patients reaching the target.

## 3. Results

### 3.1. Population Studied

A total of 113 plasma concentrations from 33 adult patients were available for this analysis. The median (min, max) number of samples per patient was three (1, 11), collected 5 h (0.13 h, 202 h) after drug intake. The administered doses ranged from 0.5 to 2 mg daily. Of the 33 patients included in this analysis, 7 (21%) were taking trametinib as monotherapy and 22 (67%) in combination with dabrafenib. Table 1 summarizes the demographic and clinical characteristics of the study population.

### 3.2. PopPK Model

A two-compartment model with linear absorption and elimination was found to best characterize the data, as the inclusion of a second compartment resulted in a significant fit improvement compared with a single-compartment model (ΔOFV = −14.41, *p* < 0.05). ka was set to a literature value of 0.913 h^−1^ and provided a median (min, max) Tmax of 1.75 h (1.51 h, 1.84 h) [38]. Introducing inter-individual variability (IIV CV%) to clearance (CL) significantly enhanced the model fit (ΔOFV = −140, *p* < 0.01), unlike the addition of IIV to the other PK parameters, which was estimated to be close to 0 (ΔOFV > −3.4, *p* > 0.05). The proportional error model was retained to characterize the residual error. The estimated parameters of the base model with IIV were a CL of 4.2 L/h (45%), a central volume (V2) of 102 L, an intercompartmental clearance (Q) of 35.1 L/h, and a peripheral volume (V3) of 338 L.

In the univariate analysis of covariates on CL, a significant association was observed with sex (ΔOFV = −12.51, *p* < 0.01), age (ΔOFV = −10.33, *p* < 0.01), BSA (ΔOFV = −7.13, *p* < 0.01), FFM (ΔOFV = −22.49, *p* < 0.01), CrCL, (ΔOFV = −11.15, *p* < 0.01), and the co-administration of dabrafenib (ΔOFV = −7.23, *p* < 0.01). The other tested covariates did not have a significant effect on CL (ΔOFV >−3.40, *p* > 0.05). In multivariate analysis, the effect of sex in addition to FFM on CL disappeared (ΔOFV > −3.40, *p* > 0.05), but the inclusion of age along with FFM resulted in a significant improvement in the model (ΔOFV = −9.20, *p* < 0.01). Likewise, the addition of co-medication with dabrafenib to FFM and age was deemed more suitable (ΔOFV = −9.00, *p* < 0.01). However, a sensitivity analysis performed on patients while monitoring their adherence revealed that the influence of dabrafenib intake on CL was no longer significant. As a result, this covariate was excluded from the final model. The effects of FFM and age remained significant in this subpopulation and were therefore retained in the final model. A positive relationship between CL and FFM was observed, resulting in a 66% increase in CL for an FFM of 68 kg, which was the maximum value observed in this study, compared with the population median of 46.35 kg. In contrast, an increase in age resulted in a decrease in CL, with a CL of 5.39 mL/h for a 30-year-old patient versus 3.22 mL/h for an 80-year-old patient (40% decrease). FFM and age explained 66% and 21% of the CL variability, respectively.

**Figure 2:**
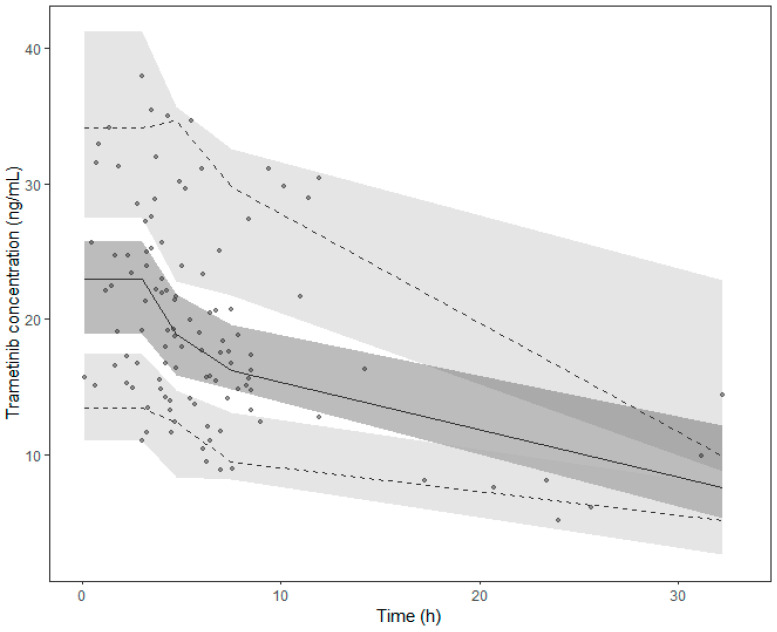
Prediction corrected visual predictive check (pcVPC) of the final model. Points collected after 35 h were excluded from the graph. Points represent the prediction-corrected observed concentrations, while the lines represent the corresponding median (solid line) and the 95% prediction interval (dashed lines). Coloured areas are the 95% confidence intervals of the model-predicted median (dark grey) and the 2.5% and 97.5% percentiles (light grey).

The final model estimations are detailed in Table 2. The internal validation via bootstrap highlighted the good precision of the model parameter estimates, as they remained within the bootstrap 95% PI and differed by less than 15% from the median parameters obtained with the bootstrap analysis (Table 2). The pcVPC supported the good predictive performances of the model (Figure 2). The goodness-of-fit plot for the final model is presented in Appendix A.

### 3.3. Simulations

**Figure 3:**
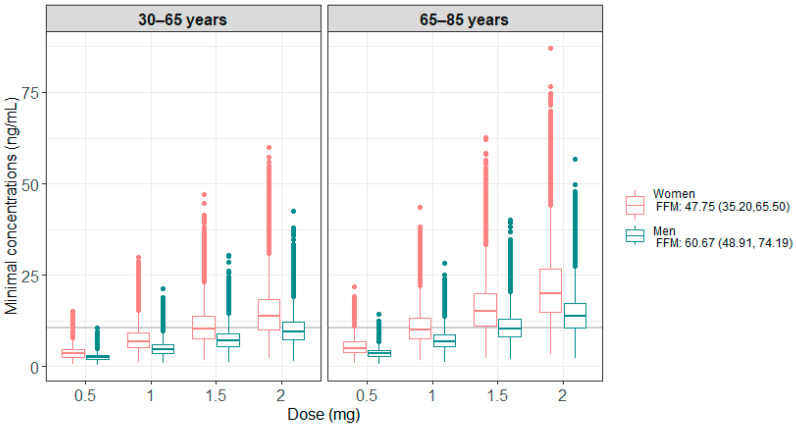
Minimum simulated concentrations obtained under the different doses tested, according to age and sex categories for all the simulations. The grey line corresponds to the target C_min_ of 10.6 ng/mL.

Figure 3 presents the results of the 100 model-based simulations performed on 4000 patients, with 1000 patients per age group (<65 years or ≥65 years) and sex. Table 3 summarizes the percentage of patients below or within the therapeutic target, i.e., C_min_ ≥ 10.6 ng/mL. These results show that men generally have lower concentrations than women and require higher doses to be within the therapeutic target. For older patients, a lower dose was found to be sufficient to reach the therapeutic target.

**Figure 4:**
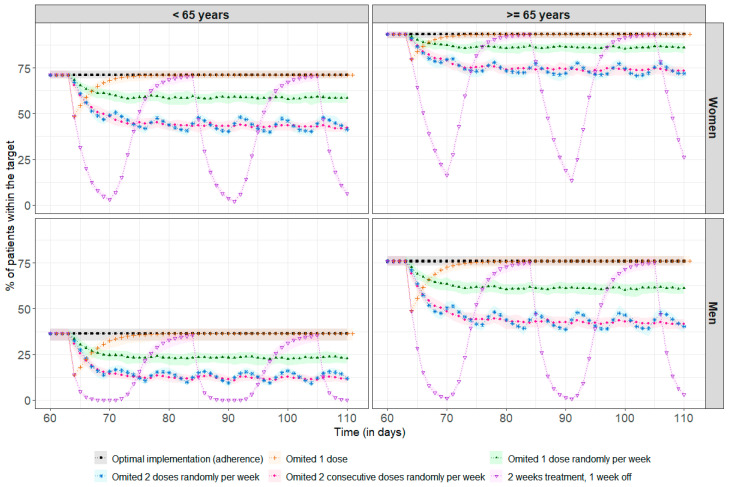
Percentage of patients reaching the therapeutic target before every drug intake (each 24 h) at steady-state (assumed here after 60 days, representing 2 months of treatment). The points correspond to the median percentage of patients within the therapeutic target at each residual time (i.e., C_min_ ≥ 10.6 ng/mL), and the coloured areas represent the min/max range obtained after 100 repetitions. The black line and squares correspond to the optimal implementation (adherence) of the drug. The orange line and crosses represent a single missed dose. The green line and solid triangles represent one randomly missed dose per week. The blue line and stars represent two randomly missed doses per week. The pink line and dots represent two consecutive missed doses per week. The purple line and inverted triangles represent treatment taken over two weeks followed by a one-week break.

Figure 4 shows the impact of poor adherence on drug exposure for a standard steady-state dose of 2 mg. Among the 69% of patients above the therapeutic target, regardless of age and sex, 31% required a median (min, max) of 3 (1, 36) days to return to concentrations above the therapeutic threshold after missing a single dose. There was no impact at all on achieving the therapeutic target in the remainder of 69%. With more frequent omissions, between once and twice a week, it is more difficult to recover steady-state concentrations, as the time between missed doses is too short compared to the required recovery time, leading to an increased risk of underdosing, with, for example, only 44% of women under 65 remaining within the therapeutic target, compared to 71% in the case of optimal adherence. After two weeks of treatment followed by a one-week break, the percentage of patients in the target range decreased significantly, especially among men under 65, with 0% of these patients remaining in the target range after three days of treatment break. The simulation results at treatment initiation are presented in Appendix B.

## 4. Discussion

This study characterizes the pharmacokinetics of trametinib in an adult cancer population, based on real-life data and using a population pharmacokinetic approach.

A two-compartment model best described the data, in line with the two previously published models of Balakirouchenane et al. and Ouellet et al. [37,38]. Due to the absence of data immediately after trametinib intake, ka could not be correctly estimated and was therefore fixed to a previously proposed value. [38]. This value yielded a median Tmax of 1.7 h, similar to the value of 1.5 h reported by the manufacturer [40]. The estimated clearances among the three popPK models were comparable, with a CL of 3.96 L/h in the current study, and 5.83 L/h and 4.91 L/h in the previous ones [37,38]. However, significant discrepancies in the central and peripheral volumes of distribution of the three models can be noted, even if all indicated an important distribution of trametinib. The present analysis allowed for the estimation of a central and peripheral volume of distributions of 108 L and 286 L, respectively, versus the 61.9 L and 417 L reported by Balakirouchenane et al. and 214 L and 568 L by Ouellet et al. [37,38]. These variations might be explained in part by the design of this current study, which is based on sparse and random sampling over 24 h after last dose. Relatively few samples were collected after 15 h, potentially introducing bias in the estimation of distribution volumes. In contrast, the two other studies included samples obtained at later time points, extending up to 100 h after the last dose administration [37,38]. The lower body weight of our population (70 (45, 96) kg) compared to the other two studies (up to 166 kg) might further contribute to the variations in volumes. Trametinib is indeed a lipophilic molecule, meaning that a higher body fat mass may lead to a more extensive distribution of the drug in the tissues, thereby generating larger volumes of distribution [40].

An analysis of covariates revealed a significant influence of fat-free mass on clearance, explaining 66% of the variability. This is the first popPK model to find this effect. Ouellet’s model reported a significant association between body weight and sex on CL [37]. These results are comparable because, in the present study, fat-free mass was estimated retrospectively using a combination of these identified factors. Currently, most anticancer treatment adjustments are based on patients’ BSA, but it has recently been shown that sex may influence these treatments [50,51] due to physiological differences between men and women [52,53]. Therefore, dosage adjustment based on fat-free mass would integrate both patients’ BSA and sex and might represent a better adjustment factor [50]. The effect of age on CL was additionally found in the present study, with older people eliminating the drug more slowly. This can be explained by a deterioration of the hepatic function with the increase in age, as well as an increase in co-morbidities and co-medications [54]. However, this effect has not been previously reported and should be confirmed by other clinical studies [37]. A 40% increase in CL in patients receiving the coadministration of dabrafenib with trametinib was identified during covariate analysis, even if the effect was poorly estimated (RSE = 46%). This finding was surprising, as a combination of the two molecules is recommended to combat the resistance induced during treatment, despite a non-clinical significant decrease in AUC reported by the manufacturer [55]. Therefore, a sensitivity analysis was performed on the subgroup of the population whose treatment history was monitored by a digital pillbox (“The full adherence information group”) and allowed for the effect of dabrafenib to be excluded. Adherence to trametinib was identified as a confounding factor in this study, leading to the misinterpretation of low concentrations seen as high clearances attributed to dabrafenib intake rather than a consequence of incorrect intake of the drug. This result emphasizes the importance of adequately providing dosing history information in popPK analyses to avoid spurious correlations.

Model simulations showed a significant difference in concentrations between men and women due to the difference in FFM between the two groups [49]. These results are consistent with clinical practice, which shows a better response to treatment but also more toxicity in women [56,57]. A comparable percentage of women and men achieved the target concentration using 1.5 mg and 2 mg daily respectively. In the same way, concentrations are higher in patients over 65 years of age, with 75% and 93% of men and women on target, respectively, compared to 37% and 71% in those under 65 years, when administered with the 2 mg dose. This highlights the potential to reduce the daily dose for patients over 65, thereby reducing adverse treatment effects.

As trametinib may lead to adverse effects that could necessitate temporary treatment discontinuation, special-case simulations were conducted to examine the impact of one or more missed doses on drug exposure. Additionally, long-term medication use may result in treatment fatigue, especially if the benefits of treatment are outweighed by the adverse effects. As a result, it is not uncommon to encounter situations where patients deliberately discontinue their medication [58]. The simulation indicates that missing one dose does not have a strong impact on concentrations, which quickly return to the concentrations obtained with an optimal intake. However, missing doses every week can become critical, especially for the subgroup of men under 65, in whom target attainment is low with the usual daily dose, hence the importance of taking the treatment correctly. It is crucial that patients receive adequate interprofessional medication adherence support, as failure to provide proper support increases the risk of non-adherence [14,59,60,61]. Several studies have experimented with programs to improve adherence, such as using a digital pillbox to help patients remember to take their medication daily, and allowing for patients and clinicians to discuss their digital results to reinforce behaviours, prevent non-adherence episodes, and promote adherence [13,62]. Factors associated with medication adherence should also be considered carefully: as shown in patients taking palbociclib, a protein kinase inhibitor used to treat breast cancer, the impact of the OpTAT pharmacist-led medication adherence intervention was larger in patients with increased disease duration and treatment experience and in patients aged >65 years [14]. In addition, more frequent visits with healthcare professionals can help manage adverse effects. Some studies have shown that increased pharmacist involvement, through appointment setting and reminder calls, is an effective way to ensure that patients take their medication as prescribed [14,62,63].

This study presents some limitations. Firstly, the popPK study was conducted on a limited number of patients. The small subsets of patients can lead to the development of a model unrepresentative of the targeted population. However, the final model characteristics converge with the preexisting models developed on a larger population. Another critical point was the difficulty in obtaining accurate information on the dosing history of the drug when self-administered, because trametinib causes several adverse effects, which can lead to treatment discontinuation. This lack of information may lead to erroneous effects on pharmacokinetic parameters. To overcome this difficulty, the history was reconstructed based on medical records, which sometimes indicated pauses in treatment. Nevertheless, there is still the possibility that the patient did not inform the clinician if a dose was missed, and the assumption of correct adherence may have slightly biased the analysis. This only affected a small number of patients, since, for a subset of the population (i.e., the “Full adherence group”), adherence to trametinib was monitored using a digital pillbox, which allowed the dynamic pattern of drug intake to be characterized. Lastly, the therapeutic target of trametinib that is currently recommended for the TDM was used to perform the simulations [18,48]. However, a higher target of 15.6 ng/mL was proposed in a study conducted on a population taking trametinib and dabrafenib [64]. The routine implementation of TDM for trametinib would allow for more personalized treatment by selecting the optimal dose for each individual patient to reach the desired therapeutic target. Nonetheless, the relationship between PK and toxicity for this drug is unclear, and increasing the dose could result in a significant increase in adverse effects [18,64,65]. The therapeutic index is so narrow that increasing the dose may not be possible. Therefore, to increase the chances of success, it is recommended that patient adherence to trametinib be monitored and supported by a medication adherence program.

## 5. Conclusions

The popPK model described drug exposure in an adult population receiving trametinib for cancer treatment and the influence of age and fat-free mass on drug elimination, highlighting the need to adjust doses according to these parameters. Further clinical studies are required to validate these findings. This study also showed that missing one or more doses per week could lead to suboptimal trametinib concentrations, with a higher risk of treatment failure, highlighting the need for appropriate interprofessional medication adherence support to optimise treatment outcomes.

## Figures and Tables

**Table 1:** Summary of patients’ characteristics.

Characteristics	n (%) or Median (min, max)	Missing Data (%)
Women ^1^	15 (45%)	-
Age [y] ^2^	63 (30, 85)	-
Body weight [kg] ^2^	70 (45, 96)	-
Size [cm] ^2^	170 (150, 188)	4
BMI [kg/m^2^] ^2^	25.3 (17.3, 33.8)	4
BSA [m^2^] ^2^	1.78 (1.40, 2.23)	4
FFM [kg] ^2^	46.35 (32, 68)	4
FFMI [kg/m^2^] ^2^	16.37 (12.96, 19.34)	4
Serum creatinine [µmol/L] ^2^	76 (46, 129)	2
CrCL [mL/min/1.73 m^2^] ^2^	84 (42, 164)	2
ASAT [U/L] ^2^	30 (12, 211)	1
ALAT [U/L] ^2^	26 (8, 152)	-
PAL [U/L] ^2^	91 (49, 873)	2
Total bilirubin [µmol/L] ^2^	5 (3, 12)	1
Type of cancer ^1^		
Melanoma	22 (67%)	-
Ovarian cancer	3 (9%)	-
Breast cancer	2 (6%)	-
Cholangiocarcinoma	2 (6%)	-
Thyroid carcinoma	1 (3%)	-
Gastrointestinal stromal tumour	1 (3%)	-
Hepatocellular carcinoma	1 (3%)	-
Ileocecal carcinoma	1 (3%)	-

Values are given according to the number of patients ^1^ or at the time of samples ^2^. BMI: body mass index, BSA: body surface area, FFM: fat-free mass, FFMI: fat-free mass index, CrCL: creatinine clearance, ASAT: aspartate aminotransferase, ALAT: alanine aminotransferase, PAL: alkaline phosphatase.

**Table 2:** Final model parameters with their bootstrap evaluations.

PK Parameter	Final Model Estimation (RSE %)	Bootstrap Performed on 2000 Runs Median (95% PI)
$k_{a}$(h^−1^)	0.913 fixed	-
$\theta_{CL}$ (L·h^−1^)	3.96 (6)	3.98 (3.52, 4.45)
$\theta_{AGE}$	−0.69 (32)	−0.66 (−1.25 to −0.20)
$\theta_{FFM}$	1.41 (17)	1.41 (0.92, 1.90)
$V_{2}$ (L)	108 (16)	101.84 (65.42, 143.40)
Q (L·h^−1^)	29.4 (30)	28.83 (12.69, 60.68)
$V_{3}$ (L)	286 (25)	286.48 (104.63, 385.40)
${IIV}_{CL}\;$(%)	23 (14)	22 (13, 28)
$\sigma_{prop}$ (%)	20 (10)	20 (16, 24)

Final equation: $CL=\theta_{CL}\times \left(1+\theta_{AGE}\times \frac{AGE-{AGE}_{median}}{{AGE}_{median}}\right)\times \left(1+\theta_{FFM}\times \frac{FFM-{FFM}_{median}}{{FFM}_{median}}\right).$ $\theta_{CL}$: typical clearance; $V_{2}$: typical volume of distribution; Q: typical intercompartmental clearance; $V_{3}$: peripheral volume of distribution; $k_{a}$: absorption rate constant; $\theta_{AGE}$: effect of age on CL (${AGE}_{median}=63$ years); $\theta_{FFM}$: effect of fat-free mass on CL (${FFM}_{median}=46.35$ kg); ${IIV}_{CL}$: inter-individual variability of CL, expressed as CV (%); $\sigma_{prop}$: proportional residual error, expressed as CV (%); 95% PI: percentile interval between 2.5% and 97.5%; RSE: standard relative error.

**Table 3:** Percentage of patients below or within the therapeutic target (i.e., 10.6 ng/mL) for each dose tested. The median and 95% confidence interval obtained after 100 replications of the simulations are shown.

Doses Tested		0.5 mg	1 mg	1.5 mg	2 mg
Age: 30–65 years					
Women	C_min_ < 10.6 ng/mL	100% [99.5%, 100%]	84% [82.1%, 87.2%]	53% [50.7%, 55.7%]	29% [26.2%, 41.5%]
	C_min_ ≥ 10.6 ng/mL	0% [0, 0.5%]	16% [12.8%, 17.9%]	47% [44.3%, 49.3%]	71% [68.5%, 73.8%]
Men	C_min_ < 10.6 ng/mL	100% [99.9%, 100%]	99% [98.3%, 99.8%]	87% [84.8%, 89.4%]	63% [59.3%, 66%]
	C_min_ ≥ 10.6 ng/mL	0% [0%, 0.1%]	1% [0.2%, 1.7%]	13% [10.6%, 15.2%]	37% [34%, 40.7%]
Age: 65–85 years					
Women	C_min_ < 10.6 ng/mL	98% [96.4%, 98.6%]	55% [51.7%, 58.3%]	21% [18.8%, 23.6%]	7% [4.8%, 8.1%]
	C_min_ ≥ 10.6 ng/mL	2% [1.4%, 3.8%]	45% [41.7%, 48.3%]	79% [76.4%, 81.2%]	93% [91.9%, 95.2%]
Men	C_min_ < 10.6 ng/mL	100% [99.8%, 100%]	90% [87.3%, 92.1%]	54% [49.6%, 57.5%]	25% [22.4%, 26.8%]
	C_min_ ≥ 10.6 ng/mL	0% [0%, 0.2%]	10% [7.9%, 12.7%]	46% [42.5%, 49.4%]	75% [73.2%, 77.6%]

**Figure A1:**
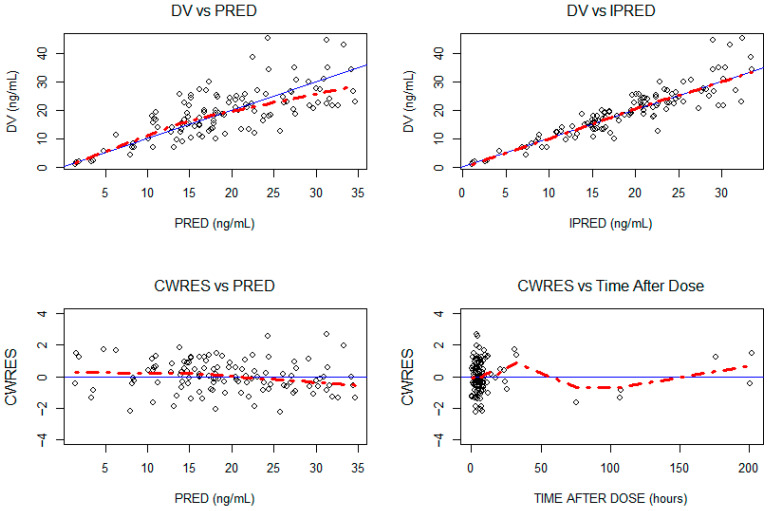
Goodness-of-fit plot for the final model. The blue line represents the Y = X identity line. The red line represents the data trend. DV: observed concentrations in ng/mL; IPRED: individual predictions in ng/mL; PRED: population predictions in ng/mL; CWRES: conditional weighted residuals.

**Figure A2:**
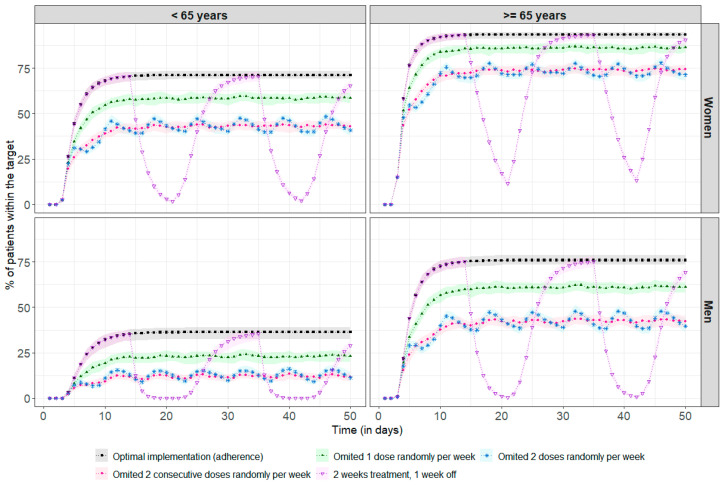
Percentage of patients reaching the therapeutic target every drug intake (each 24 h). The points correspond to the median percentage of patients within the therapeutic target at each residual time (i.e., C^min^ ≥ 10.6 ng/mL), and the coloured areas represent the min/max range obtained after 100 repetitions. The black line and squares correspond to the optimal implementation (adherence) of the drug. The green line and solid triangles represent one randomly missed dose per week. The blue line and stars represent two randomly missed doses per week. The pink line and dots represent two consecutive missed doses per week. The purple line and inverted triangles represent treatment taken over two weeks followed by a one-week break.

## Data Availability

The data presented in this study are available on request from the corresponding author. The data are not publicly available due to ethical reasons.

## Abbreviations

ALAT	Alanine aminotransferase
ASAT	Aspartate aminotransferase
BFM	Body fat percentage
BMI	Body mass index
BSA	Body surface area
BW	Bodyweight
CL	Clearance
C_min_	Minimum concentration
CrCL	Creatinine clearance
CYP	Cytochrome P
FFM	Fat-free mass
IIV	Inter-individual variability
ka	Absorption rate constants
MAPK/ERK	Mitogen-activated protein kinase/extracellular-regulated kinase
MEK	Mitogen-activated protein kinases
Nonmem	Nonlinear mixed effect modelling
OpTAT	Optimizing oral Targeted Anticancer Therapies
PAL	Alkaline phosphatase
pcVPC	Prediction-corrected visual predictive check
PK	Pharmacokinetics
popPK	Population pharmacokinetic
PsN	Pearl speaks Nonmem
Q	Intercompartmental clearance
RSE	Relative standard error
TDM	Therapeutic drug monitoring
Tmax	Time required to reach maximum concentration
V2	Central volume
V3	Peripheral volume
ΔOFV	Difference in objective function values
95% PI	95% prediction interval
